# An Unexpected Cause of Fever After Weaning From Veno-Arterial Extracorporeal Membrane Oxygenation (vaECMO): A Case Report

**DOI:** 10.7759/cureus.82278

**Published:** 2025-04-14

**Authors:** Christina Hadjilouca, Christos Efseviou, Antonia Kastoris, Anna Vavlitou

**Affiliations:** 1 Intensive Care Unit, Nicosia General Hospital, Nicosia, CYP

**Keywords:** ecmo complications, menstrual cup complications, retained menstrual cup, unexplained fever, va-ecmo

## Abstract

Fever following decannulation from extracorporeal membrane oxygenation (ECMO) poses diagnostic challenges. While infectious causes and thrombosis are typically considered, rare etiologies may be overlooked. We report the case of a 47-year-old woman who developed a high, refractory fever following veno-arterial (va) extracorporeal membrane oxygenation decannulation. Extensive infectious and non-infectious workups failed to identify a definitive cause as cultures, imaging modalities, and inflammatory markers were inconclusive. On post-ECMO day 9 and 23 days after initial admission to the hospital, a gynecological examination was prompted by unexplained unilateral labial edema and prolonged menstruation. Speculum examination revealed a retained menstrual cup embedded in the cervix, causing local inflammation. Following its removal, the patient’s fever rapidly resolved, without the need for further antibiotic escalation. Menstrual cups are generally considered safe, with rare complications such as toxic shock syndrome, pelvic infections, and mechanical injuries. However, in critically ill patients unable to provide a complete history, retained menstrual cups can lead to significant inflammatory responses and diagnostic delays. This case highlights the importance of maintaining a broad differential diagnosis, including gynecological evaluation, in cases of unexplained fever, particularly in critically ill patients.

## Introduction

Extracorporeal membrane oxygenation (ECMO) is an advanced life-support modality that provides temporary extracorporeal support for gas exchange and/or circulatory function in patients with severe acute respiratory or circulatory failure. It is utilized as a therapeutic intervention in cases refractory to conventional management, serving as a bridge to recovery, transplantation, or long-term mechanical support. Despite its life-saving potential, ECMO is a complex modality associated with various complications, which may arise during the course of therapy or after weaning.

Fever is a common occurrence following ECMO decannulation and can pose significant diagnostic challenges. Studies indicate that around half of post-ECMO patients develop fever [1], which may result from infectious causes - such as bloodstream infections, pneumonia, or catheter-related infections - or non-infectious etiologies, including thrombosis or drug reactions [2-4]. However, in some cases, the cause of fever after decannulation from ECMO remains elusive, prompting clinicians to consider less common etiologies.

In a retrospective, single-center study involving 123 patients successfully weaned from veno-arterial (va) or veno-venous (vv) ECMO support, Assouline et al. found that fever typically occurred within the first 24 hours following decannulation and could persist for up to five days, regardless of the identified cause. Notably, while patients with infections experienced prolonged mechanical ventilation and longer ICU stays, mortality rates did not significantly differ between those with infectious and non-infectious causes of fever. Infections, namely ventilator-associated pneumonia (VAP), bloodstream infections, and soft tissue infections, were diagnosed based on microbiological confirmation of the pathogen in appropriate cultures within the context of relevant clinical findings, whereas thrombosis was identified by ultrasound or computed tomography (CT). In 17% of patients who developed fever post-decannulation, no clear underlying cause as stated above was identified. Of note non-standard causes of fever, including gynecological sources of infection, were not considered [1]. In these cases, fever is likely multifactorial, although the precise pathophysiology of post-decannulation fever remains poorly understood. 

A contributing factor to this phenomenon may be the exposure of the patient’s blood to the non-endothelialized surface of the ECMO circuit, which can trigger an inflammatory response, similar to that seen in extracorporeal circulation [5]. Thangappan et al. reported that systemic inflammatory response syndrome (SIRS), defined in their study by at least two of the following criteria, fever, leukocytosis, and escalation of vasopressor support, developed in 60% of patients after ECMO decannulation. Notably, 40% of these cases could not be attributed to infection [6].

Despite the potential role of inflammatory responses, fever following decannulation should not be considered a benign reaction without a thorough diagnostic work-up to exclude infection and thrombosis, as these conditions can have devastating consequences, such as sepsis, embolism, or ischemia, and carry significant therapeutic implications.

## Case presentation

A 47-year-old woman, mother of four, with no significant past medical history, not currently using contraception, and a non-smoker, presented with acute, severe retrosternal chest pain radiating to her left arm, lasting for 30 minutes. She was transported by ambulance to a peripheral hospital, where her electrocardiogram (ECG) and troponin levels were consistent with a non-ST-elevation myocardial infarction (NSTEMI). That night, she experienced a recurrent episode of chest pain, followed by ventricular fibrillation (VF) cardiac arrest, which was successfully treated with external electrical defibrillation. A repeat ECG demonstrated anterior ST-elevation myocardial infarction (STEMI), prompting urgent transfer to a tertiary center for emergency coronary angiography.

**Figure 1:**
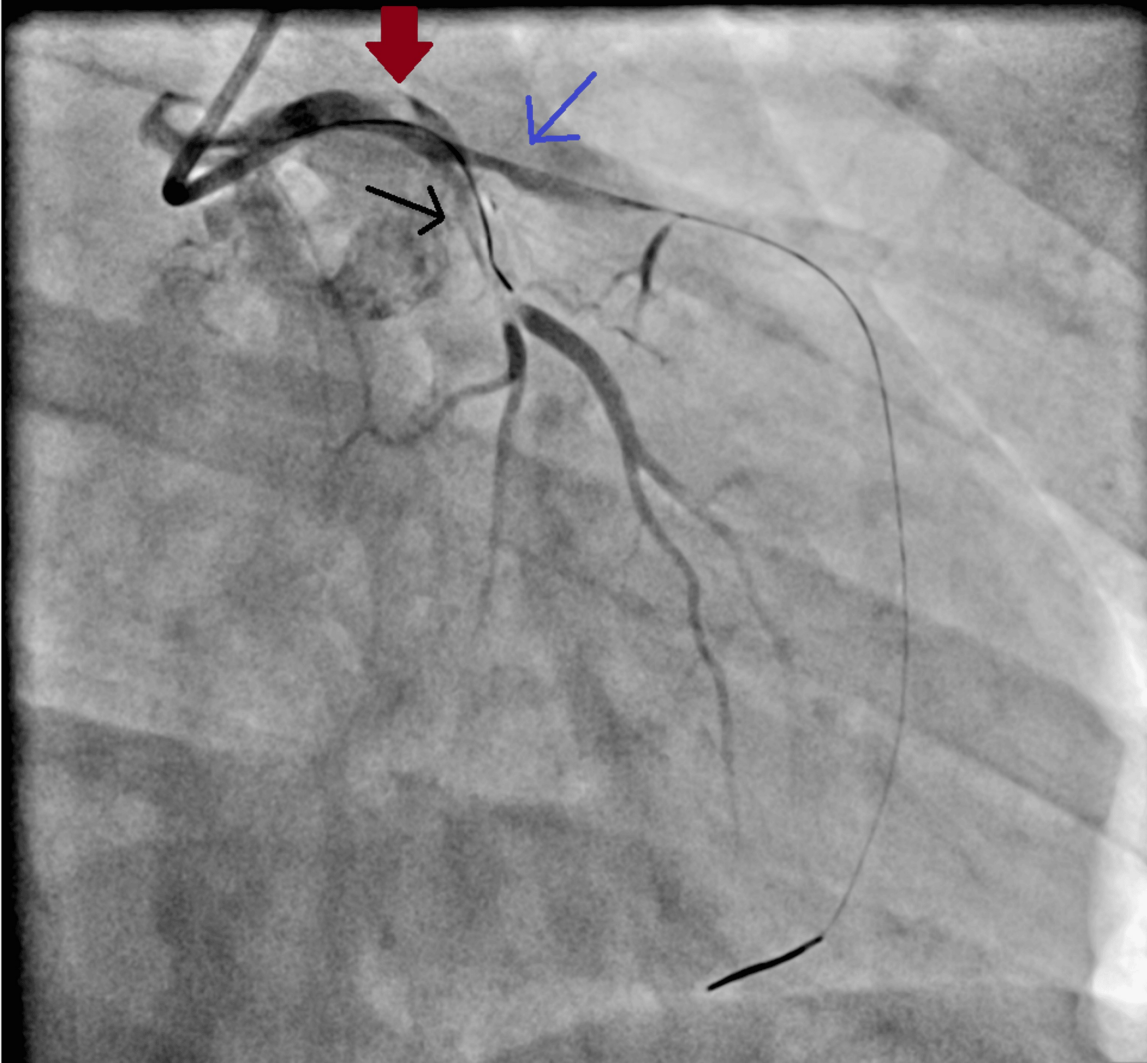
Coronary angiography Dissection of the left main coronary artery (LMCA) (red arrow), extending into and obstructing flow in both the left anterior descending (LAD) artery (blue arrow) and the left circumflex (LCX) artery (black arrow).

Coronary angiography, performed approximately three hours after the recurrence of chest pain, revealed left main coronary artery (LMCA) dissection extending into and obstructing the left anterior descending (LAD) and left circumflex (LCX) arteries (Figure 1).

During the procedure, the LMCA dissection led to worsening hemodynamic instability, necessitating the insertion of an intra-aortic balloon pump (IABP) and immediate transfer to the operating room for emergency coronary artery bypass grafting (CABG).

**Figure 2:**
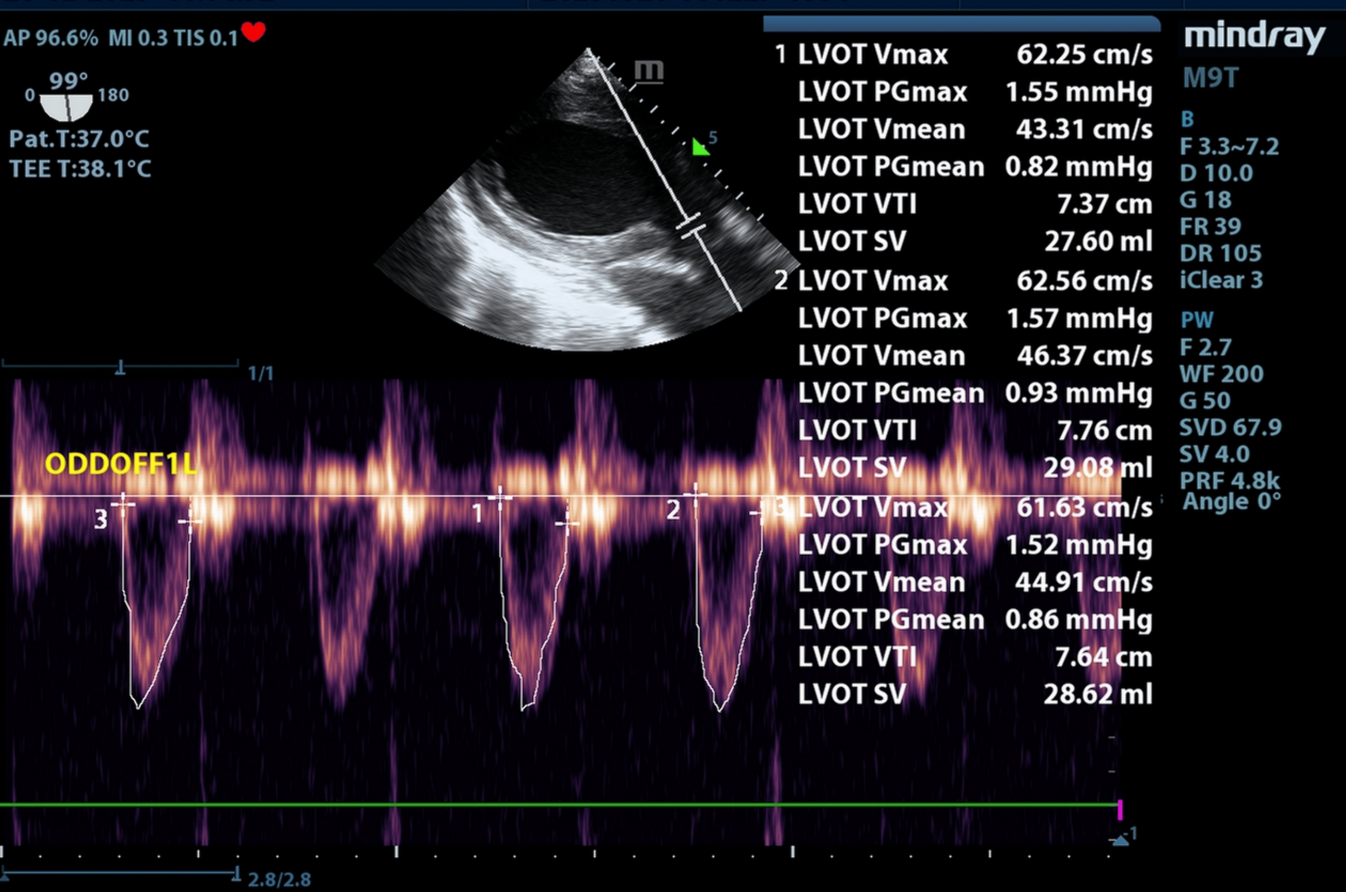
Transgastric long-axis view Pulse wave (PW) Doppler at the left ventricular outflow tract (LVOT), used to calculate LVOT velocity time integral (VTI) and stroke volume (SV). The heart rate is 75 bpm, with a calculated SV of 28.7 mL and a cardiac output (CO) of 2.1 L/min. These findings are consistent with low CO and cardiogenic shock.

Upon anesthesia induction, she suffered pulseless electrical activity (PEA) cardiac arrest. Urgent sternotomy was performed, and cardiopulmonary resuscitation (CPR) was initiated. The patient was placed on cardiopulmonary bypass, and a successful CABG was performed (LIMA to LAD, SVG to OM). However, weaning from cardiopulmonary bypass was unsuccessful due to severe left ventricular (LV) dysfunction (Video 1) (Figure 2), necessitating central vaECMO. 

**Video 1:** Transesophageal long-axis view Long-axis view of the left ventricle showing akinesis of the mid-anterior septum, mid-inferolateral wall, and apex.

After three days of intensive care unit (ICU) stabilization, the patient remained ECMO-dependent, prompting conversion to a semi-peripheral vaECMO configuration with a subclavian artery return cannula and femoral vein drainage cannula. Following an uneventful ICU course with supportive care, including levosimendan infusion, she was deemed suitable for ECMO weaning on day 13. At that point, she had undergone tracheostomy, sedation had been discontinued, and she was awake and communicative. However, she had not yet been mobilized out of bed and remained dependent on mechanical ventilation. 

The day prior to ECMO removal, the patient developed a moderate fever, reaching 38.5°C, despite ongoing ECMO support. Following successful ECMO weaning, her hemodynamic and cardiac status remained stable; however, she developed a high-grade fever (up to 40.1°C) that persisted over several days. The fever pattern was intermitted, with evening and nocturnal peaks, and no complete resolution during the daytime. Remarkably, she remained clinically well throughout these episodes, without rigors, hypotension, or altered consciousness. Clinical examination was unremarkable, except for increased bronchial secretions and generalized anasarca, including edema of the external genitalia. Inflammatory markers, including C-reactive protein (CRP) and procalcitonin, were only mildly elevated, while the white blood cell (WBC) count was moderately increased (Table 1).

**Table 1:** Inflammatory markers The course of inflammatory markers following ECMO decannulation. CRP and procalcitonin remained relatively low but there was a moderate increase of WBC. WBC, white blood cells; CRP, C-reactive protein; N/A, not available

	ECMO decannulation	Post-ECMO day 3	Post-ECMO day 7	Reference ranges
CRP (x10^3^/μ)	74.7	36.9	46.3	<5
WBC (x10^3^/μ)	12.62	21.03	15.18	4.37-9.68
Procalcitonin (ng/mL)	N/A	0.27	0.39	<0.50

These results, along with negative microbiological findings, were considered inconclusive, as mild to moderate fluctuations in inflammatory markers are commonly observed in critically ill patients undergoing multiple interventions.

**Figure 3:**
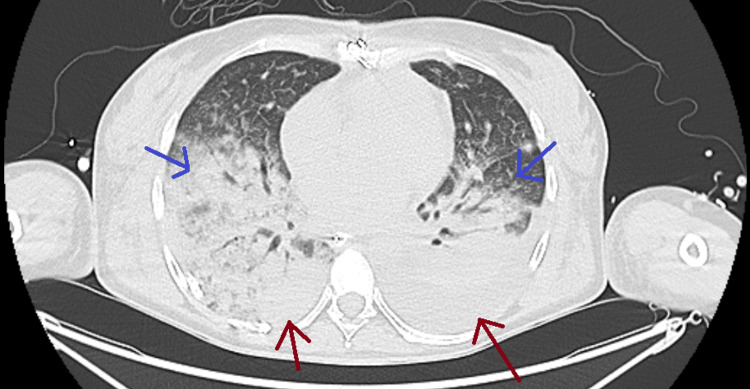
CT scan of the lungs Bilateral pleural effusions (red arrows) and basal atelectasis/consolidations (blue arrows). CT, computed tomography

A comprehensive infectious workup was initiated: blood, bronchial, and urinary cultures were obtained on the first day of fever and repeated every two to three days, all of which remained negative. Replacement of the central venous catheter, arterial line, and urinary catheters had no impact on the fever pattern. A CT scan of the thorax and abdomen on day 4 post-ECMO revealed bilateral pleural effusions and basal atelectasis/consolidations, but no evidence of mediastinitis or intra-abdominal abscess (Figure 3).

Bronchoscopy with bronchoalveolar lavage (BAL), performed on day 5 post-ECMO, as well as pleural fluid analysis obtained during the same period showed no microbiological signs of infection. Cardiac ultrasound excluded endocarditis or pericarditis, but a thrombus in the inferior vena cava (IVC) was observed. Therapeutic anticoagulation was initiated for the IVC thrombus, which was closely monitored and showed gradual resolution.

Empirical antibiotic therapy was escalated stepwise, from ceftriaxone and vancomycin to piperacillin-tazobactam, then meropenem, and ultimately colistin, in an effort to target potential multidrug-resistant gram-negative organisms commonly encountered in our ICU. Despite this broad-spectrum coverage, the fever persisted without resolution.

Given the persistence of fever despite the exclusion of profound infectious causes, non-infectious etiologies were systematically evaluated. All non-essential medications were discontinued to address possible drug-induced fever, although no associated eosinophilia was observed. Neuroleptic malignant syndrome was ruled out, as the patient had not received any related medications such as antipsychotics and antiemetics, and there were no signs of muscular rigidity or rhabdomyolysis. An autoimmune etiology was also considered but deemed unlikely, as screening tests, including antinuclear antibodies (ANA), anti-double stranded DNA (anti-dsDNA) antibodies, anti-Smith (anti-Sm) antibodies, rheumatoid factor (RF), and antineutrophil cytoplasmic antibodies (p-ANCA and c-ANCA), were all negative during the initial workup for spontaneous coronary artery dissection upon ICU admission. The possibility of a non-infectious post-ECMO fever, typically regarded as a diagnosis of exclusion, was also considered. However, the persistence of high-grade fever several days after decannulation made this diagnosis less likely.

**Figure 4:**
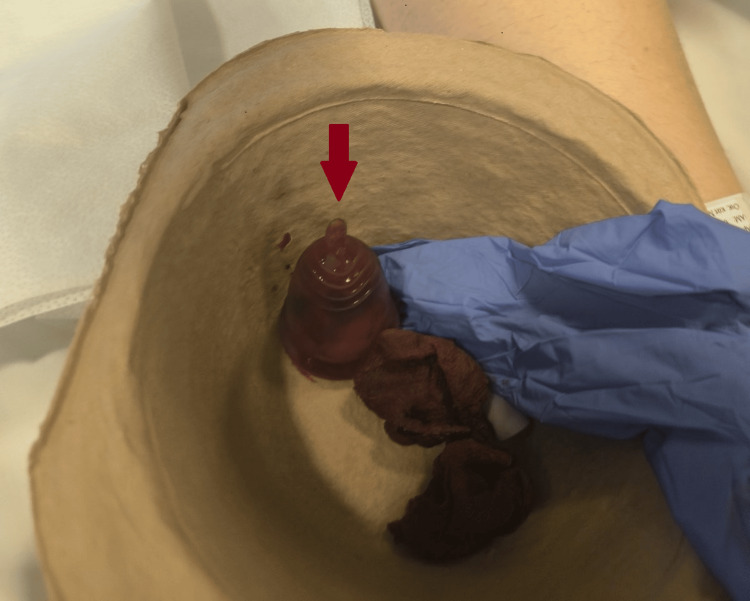
Menstrual cup The red arrow indicates the menstrual cup after its removal from the patient's cervix.

On post-ECMO day 9 and 23 days after initial hospital admission, a gynecological examination was requested due to worsening labial edema, which had become unilateral and could no longer be fully attributed to generalized anasarca. This was accompanied by prolonged menstrual bleeding. Speculum examination revealed a foreign object embedded in the cervix, associated with local inflammation extending to the vagina and external genitalia, though no purulent discharge was observed. Upon removal, it was identified as a forgotten menstrual cup (Figure 4).

That same afternoon, the fever began to subside, and by the following day, she was afebrile. At the time, she was receiving broad-spectrum antibiotic therapy, including vancomycin, meropenem, and colistin. Subsequently, the cervical swab cultures identified Acinetobacter baumannii, a multi-drug resistant organism, which was found to be resistant to both meropenem and colistin. However, by this point, the patient had already become afebrile and clinically stable, and therefore, further escalation of antibiotics was not deemed necessary. 

**Figure 5:**
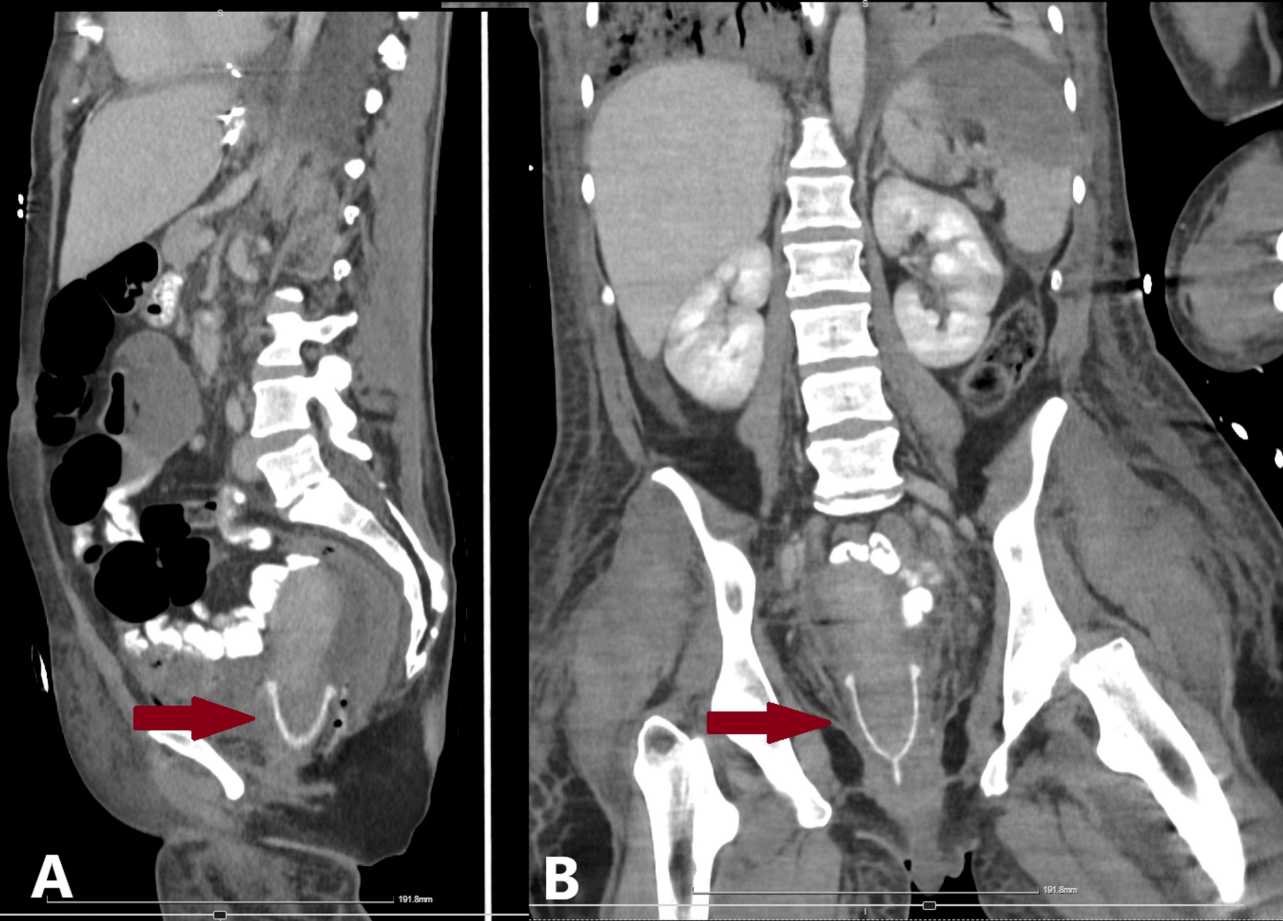
CT of the abdomen and pelvis. (A) Sagittal view; (B) Coronal view Visible menstrual cup located at the cervix (red arrows). CT, computed tomography

In light of the gynecological findings, a retrospective review of the CT scan performed several days earlier revealed the presence of a menstrual cup positioned at the cervix (Figure 5). This had been overlooked in the initial report, likely due to a combination of low clinical suspicion and unfamiliarity with such devices. 

## Discussion

Menstrual cups are a reusable alternative to conventional tampons, designed to collect rather than absorb menstrual flow. They are made of medical-grade materials such as silicone, rubber, latex, or elastomer and can last for up to 10 years. With a capacity of up to 40 mL, they should typically be emptied every four to twelve hours, depending on menstrual flow and cup type [7].

The use of menstrual cups is generally considered safe, with relatively few adverse effects reported in the literature. The most commonly described complications include vaginal wounds, vaginal or cervical irritation without significant clinical consequences, and allergic reactions [7]. However, these adverse effects are primarily associated with typical use in otherwise healthy individuals. Limited data exist regarding potential complications in critically ill patients, particularly in cases where the presence of a menstrual cup goes unrecognized.

Menstrual toxic shock syndrome (mTSS) is a severe, multisystem, toxin-mediated disease primarily associated with tampon use. It is characterized by fever, rash, hypotension, and multi-organ failure, which can be fatal if not promptly recognized and treated. However, the case presented above differs significantly. The patient appeared clinically well despite a high-grade fever, showing no signs of toxicity, coagulopathy, hypotension, or other systemic involvement. Furthermore, the microorganism identified by the cervical swab culture was resistant to antibiotics administered at the time, and the fever resolved rapidly following the removal of the menstrual cup, without further antimicrobiological agent escalation. These observations suggest a pronounced local inflammatory response to the retained foreign body, rather than a case of infection or mTSS.

While menstrual cups are believed to pose a lower risk of mTSS compared to tampons due to their composition, an in-vitro study assessing the impact of menstrual cups on Staphylococcus aureus growth and toxic shock syndrome toxin-1 (TSST-1) production found that menstrual cups facilitated higher levels of S. aureus proliferation and toxin production than tampons. This finding has been attributed to the cup’s shape, which allows for increased air exposure, a known factor in S. aureus growth and TSST-1 production [8]. Nevertheless, these findings remain theoretical and have not been validated in clinical trials.

To date, a few confirmed cases of menstrual cup-associated toxic shock syndrome (TSS) have been reported, three of which are case reports [9-11]. The first case, documented in 2016 in a 37-year-old woman, met diagnostic criteria for TSS despite lacking microbiological confirmation [9]. In a 2020 case, a patient infected with methicillin-sensitive S. aureus (MSSA) producing TSST-1 developed acute respiratory distress syndrome (ARDS) and septic cardiomyopathy, requiring intubation, mechanical ventilation, and vasopressor support [10]. The third case, which was also microbiologically confirmed, involved a patient whose genital cultures grew MSSA; she was also admitted to the ICU and required vasopressor therapy [11]. All three cases had favorable courses following appropriate treatment with patients discharged well.

In another case, a 42-year-old female patient presented with fever, abdominal pain, and shortness of breath and was diagnosed with concurrent pneumonia and pelvic peritonitis. She was found to have been using a menstrual cup on the fifth day of her menstrual cycle. Following the removal of the cup, emergency exploratory laparoscopy and right ovariectomy were performed due to suspected peritonitis. Streptococcus pneumoniae was isolated from bronchoaspirate and intraoperative peritoneal fluid cultures, though the primary source of infection remained unclear. The patient developed hemodynamic instability requiring vasopressor support, mechanical ventilation, and eventually right lower lobectomy due to necrotizing pneumonia. She ultimately recovered and was discharged in stable condition [12].

Mechanical complications from the menstrual cup have also been reported. One case involved a patient presenting to a genitourinary clinic with a retained menstrual cup that had been inserted the previous night. On speculum examination, the cup was visualized high in the vagina, with the cervix firmly lodged within it, making retrieval difficult despite adherence to product instructions [13]. Additionally, six cases of hydronephrosis or ureterohydronephrosis associated with menstrual cup use have been reported [14-19]. These patients presented with renal colic, and imaging studies (ultrasonography or CT scan) revealed hydronephrosis or ureterohydronephrosis, with no evidence of obstructive causes such as lithiasis. In all cases, symptoms and imaging abnormalities resolved following the removal of the improperly positioned menstrual cup, indicating that mechanical pressure from the device, and possibly associated local inflammation, were responsible for the obstruction. This mechanism may likewise contribute to mechanical or inflammatory complications in critically ill patients with a retained menstrual cup, as demonstrated in the present case. 

To the best of our knowledge, there are no reported cases of forgotten or incidentally discovered menstrual cups in hospitalized patients with impaired communication, such as those in critical care settings. In these circumstances, a menstrual cup may go unnoticed, as it is not typically visible during routine clinical examination or standard nursing care. If retained for prolonged periods, the cup can act as a foreign body, potentially causing local or systemic inflammation. Gynecological examinations are infrequently performed in critically ill patients, even if unexplained fever is present, as other diagnoses are typically prioritized and clinical suspicion remains low in the absence of clear signs or relevant patient history. This delay in recognition can result in prolonged retention, increasing the risk of complications such as local and systemic inflammation.

Although menstrual cups are generally composed of materials such as silicone, rubber, or elastomer, which are typically non-radioopaque, their visibility on CT scans may vary depending on the specific composition and the presence of additives. Notably, as in our case, in all the cases presented above, the device was clearly visible on CT scans [12,14,16-19] or abdominal X-rays [15] where performed. This suggests that missed diagnoses may result from a lack of clinical suspicion, compounded by the potential unfamiliarity of the reporting physician with the existence and appearance of such devices, rather than a lack of radioopacity.

## Conclusions

Persistent fever in post-ECMO patients presents a significant diagnostic challenge. A comprehensive full-body clinical examination, including gynecological assessment when appropriate, should be performed early, repeated regularly, and used to guide targeted investigations. While infection and thrombosis remain the most common causes, less typical etiologies, such as retained foreign bodies causing local sterile or infectious inflammation, must also be considered. In this context, diagnostic accuracy relies not only on laboratory and imaging results but also on their careful interpretation in conjunction with clinical findings. Furthermore, imaging reviews should be thorough, excluding both common and less frequently encountered diagnoses, as demonstrated in this case. This approach is particularly crucial in all critically ill, sedated, or non-communicative patients who are unable to report symptoms or provide a reliable history. Applying a structured, examination-oriented strategy can improve diagnostic yield and avoid delays, making it a valuable framework in the evaluation of unexplained fever in ICU patients. This case underscores the importance of thorough clinical assessment and maintaining a broad differential diagnosis in the management of persistent fever in the critically ill.
